# Atrial Fibrillation in Heart Failure: Epidemiology, Quality of Life and Clinical Characteristics in the Iron Deficiency and ANaemia in Heart Failure (IDAN-HF) Study in Ogbomoso, Nigeria

**DOI:** 10.4314/ejhs.v33i2.9

**Published:** 2023-03

**Authors:** Adeseye A Akintunde, Olawale M Akinlade

**Affiliations:** 1 Department of Medicine, LAUTECH Teaching Hospital, Ogbomoso, Nigeria; 2 Goshen Heart Clinic, Osogbo, Osun State, Nigeria; 3 Department of Medicine, Faculty of Clinical Sciences, Ladoke Akintola University of Technology, Ogbomoso, Oyo State, Nigeria; 4 Cardiology Department, Royal Infirmary of Edinburgh, 51 Little

**Keywords:** Atrial fibrillation, heart failure, epidemiology, clinical characteristics, Southwestern Nigeria

## Abstract

**Background:**

The cluster of atrial fibrillation (AF) with heart failure (HF) may be associated with a poorer prognosis. Its epidemiology and impact on clinical outcomes and quality of life among HF subjects in Africa have not yet been fully described. This study aimed at describing the epidemiology of AF among HF subjects, its impact on quality of life, clinical characteristics, and associations.

**Methods:**

140 HF subjects were recruited by stratified random sampling method, and 12-lead electrocardiography was done to diagnose AF. Statistical analysis was done with SPSS 21.0. Informed consent was obtained from all participants.

**Results:**

The frequency occurrence of AF was 28 (20.0%) of the HF subjects and were similar in age, systolic blood pressure, diastolic blood pressure, right ventricular internal dimension, packed cell volume, and gender distribution with those in sinus rhythm. The mean (S.D) six minutes walk test distance was significantly lower among HF subjects with AF compared to those without AF (171.1± 88.9 vs. 225.8 ± 102.1m respectively, p <0.05). Pulmonary hypertension, intracardiac clots, and kidney dysfunction were more frequent among HF subjects with AF than among those without AF. HF subjects with AF had a higher frequency of clusters of comorbidities than those without AF. AF was most prevalent and left atrial dimension was highest among subjects who had HF with reduced ejection fraction, compared to other HF phenotypes.

**Conclusion:**

AF is common in HF among Nigerians and is associated with poor quality of life and poorer functional status compared to those with sinus rhythm.

## Introduction

Atrial fibrillation (AF) and heart failure (HF) frequently coexist together or in clusters with other comorbidities (1). AF is the commonest cardiac arrhythmia, and it is common in the elderly (2,3). The coexistence of AF with HF may be associated with a poor prognosis, worse clinical and functional outcomes, and increased healthcare expenditure (2,4). Stable HF may often be escalated into acute decompensation by cardiac arrhythmias, including AF, with a further risk of cardiac embolism (5). The loss of atrial kick in AF denies the heart of atrial supplementation in every cardiac cycle, and this by itself can precipitate HF or enhance other risk factors to lead to rapid cardiovascular decompensation (6,7).

The epidemiology of AF and its impact on clinical outcomes and quality of life among HF subjects is not yet fully described among Africans. AF among Africans is more likely to be valvular AF due to a high burden of rheumatic and infective valvular heart diseases (8–10). Some authors have shown that AF is associated with advanced HF and certain other clinical characteristics, including age, gender, and HF phenotype, among others (11,12).

There is a complex interplay between HF and AF. HF can precipitate AF, and AF can also lead to HF (5,11,13–16). The impact of AF in HF subjects should therefore be of major interest among Africans due to the variability in the aetiology of AF between Africans and Caucasians. It is important to study the specific impact and variations in the clinical characteristics and quality of life caused by each of these conditions on the other and to reduce the morbidity and mortality associated with HF in sub-Saharan Africa. We, therefore, sought to describe the epidemiology of AF among HF subjects in the Iron Deficiency and ANemia in Heart failure (IDAN-HF) study, its impact on quality of life, clinical characteristics, and associations in Ogbomoso, southwest Nigeria.

## Methods

This study was conducted in the cardiology clinics of two teaching hospitals in Ogbomoso, southwest Nigeria—Ladoke Akintola University of Technology Teaching Hospital, Ogbomoso, and Bowen University Teaching Hospital. One hundred and forty chronic HF subjects who were being seen at the study centers and had been on follow-up for at least six months were recruited through a stratified random sampling method. The diagnosis of HF was made using the Framingham criteria (17). The patients were recruited over six months. The full details of the methodology for the Iron Deficiency and ANaemia in Heart Failure (IDAN-HF) study have been published elsewhere (11). The clinical data and other information of enrolled participants were recorded with a data collection form. The data obtained include age, gender, occupation, marital status, address, tribe, history of hypertension and diabetes mellitus, smoking, and alcohol intake. Systolic and diastolic blood pressures were measured according to standardized guidelines after patients had been seated for at least five minutes. Patients were included in the study if they fulfilled the inclusion criteria, which included having been treated and followed up for HF for at least six months, being≥18 years of age, and having given written informed consent to participate in the study. Patients were excluded from the study if they were pregnant, if they had other chronic diseases for which they were receiving treatment (such as malignancy, chronic kidney disease requiring dialysis, or major psychiatric illnesses), or if they had had acute decompensated HF in the previous two weeks.

All participants were thoroughly examined clinically. Weight was measured with a standardized weighing scale to the nearest 0.5 kg. Height was measured with a stadiometer to the nearest cm. Body mass index was determined and categorized as normal, overweight, mild obesity, moderate obesity, and severe obesity. Various investigations were done, which include full blood count, blood film appearance, full iron parameters (including total iron, serum ferritin, transferrin saturation, and total iron binding capacity, to determine iron deficiency status), urinalysis, and electrolytes, urea and creatinine. Echocardiography was done according to the American Society of Echocardiography guidelines, with the patient in the left lateral position. Anaemia was defined as Hb <12g/dl in women and <13g/dl in men according to the World Health Organisation (WHO) criteria. Atrial natriuretic peptides (ANP) assay was done using the Elabscience enzyme-linked immunosorbent assay (ELISA) kit method (Catalog No: E-EL-H0532). The normal range of ANP was defined as 86–111 pg/ml according to the manufacturer's manual. Pulmonary hypertension was defined as estimated pulmonary arterial systolic pressure greater than 35mmHg. The Pulmonary arterial systolic pressure was evaluated with the addition of tricuspid regurgitant velocity gradient to the estimated right atrium diastolic end-diastolic pressure. Iron deficiency was defined as Transferrin saturation (TSAT) <20% because TSAT is a better marker of iron status than ferritin during inflammation as ferritin overestimates iron store in inflammatory conditions.

Transferrin saturation (TSAT) was determined as 100 x serum iron/TIBC. A 12-lead electrocardiogram was done for all participants according to **a** standardized protocol. The interpretation was done by the two researchers who are senior cardiology fellows, and they were blinded to the clinical data during electrocardiogram interpretation. The parameters measured include heart rate, mean QRS axis, PR interval, P wave duration, and amplitude, QRS wave duration and morphology, QTc intervals, ST segment for changes in depression or elevation, T wave abnormalities, and Electrocardiography (ECG) heart chamber enlargement. AF was defined as the absence of a P wave on routine 12-lead ECG with or without irregular R-R interval. AF is a type of supraventricular tachyarrhythmia and it is the commonest type. Long rhythm strips were used often in making the diagnosis. Intracardiac clot(s) refer to suspected clots in at least one chamber of the heart that was found on standard transthoracic echocardiography. Further imaging could not be done to confirm the presence of intracardiac clots due to the non-availability of cardiac magnetic resonance (CMR), contrast echocardiography, or transesophageal echocardiography. Anticoagulation in these patients was estimated to be poor as most of them were on very low doses of warfarin at study entry with no serial monitoring of the international normalized ratio due to financial constraints.

Ethical approval was obtained from the Research Ethics Committee of LAUTECH Teaching Hospital, Ogbomoso, Nigeria. All participants gave their written informed consent. Statistical analysis was done with the Statistical Package for Social Sciences version 21.0 (Chicago Ill, IBM). Qualitative variables were summarized as frequencies (percentages), while quantitative variables were summarized as means (standard deviation). Student's t-test, analysis of variance, or Chi-square test were used as appropriate, to determine the statistical significance of the difference between the two groups. P <0.05 was taken as statistically significant.

**Ethical Approval:** Ethical approval was obtained from the Research Ethics Committee of Ladoke Akintola University of Technology Teaching Hospital, Ogbomoso, Nigeria (approval number LTH/2018/075). Informed written consent was obtained from all participants.

## Results

The mean age of study participants was 62.96 ± 16.3 years. Mean (S.D) systolic and diastolic blood pressures were 125.1 ± 22.5 mmHg and 78.23 ± 14.0 mmHg respectively. The mean (S.D) heart rate was 90.2 ± 18.8 beats per minute, while the mean body mass index (S.D) was 24.7 ± 6.5 kg/m2. There were more females among the study participants (80, 57.1%), and 87.1% of all study participants were previously diagnosed to have hypertension. Mean (S.D) serum urea and creatinine were 17.2 ± 22.7 µmol/l and 126.7 ± 117.4 mmol/l respectively as shown in Table 1.

**Table 1:** Clinical characteristics of study participants with and without atrial fibrillation

Variable	All	AF (n=28)	Sinus rhythm (n=112)	P value
Age, years	63.0 ± 16.3	60.8 ± 15.3	63.5 ± 16.6	0.426
SBP, mmHg	125.1 ± 22.5	123.4 ± 26.3	125.6 ± 21.5	0.645
DBP, mmHg	78.2 ± 14.0	80.4 ± 17.5	77.7 ± 13.0	0.355
FBS, mmol/l	6.7±2.1	8.4 ± 2.0	5.4 ± 0.8	0.001*
LAD, mm	47.4±11.6	54.5 ± 8.5	45.5 ± 11.6	0.001*
RVD, mm	30.3±5.1	31.8 ± 4.2	29.9 ± 5.2	0.110
EF, %	40.7±9.4	37.7± 5.4	44.5 ± 10.1	0.049*
LVMI, g/m^ht^	68.8±47.4	103.1 ± 42.7	60.2 ± 44.7	0.000*
KCCQ, %	66.4±15.4	64.9 ± 17.7	66.7 ± 14.8	0.482
6MWT distance, mm	216.8±101.6	171.1± 88.9	225.8 ± 102.1	0.046*
Gender, male/female, n	60(42.9%)/80 (57.1%)	15/13	45/67	0.200
Pulmonary HTN, n, %	48(34.3%)	18 (64%)	30 (26%)	0.000*
Intracardiac clots, n %	39(27.9%)	16 (57%)	23 (20%)	0.022*
NYHA IV, n, %	43(30.7%)	14 (50.0%)	29 (25.0%)	0.049*
HTN, n, %	122 (87.1%)	25 (89.0%)	97 (86.0%)	0.705
Mean Hb (g/dl)	10.6±2.5	10.8±2.9	11.1±2.4	0.545
PCV,%	32.2 ± 6.4	31.6 ± 7.9	32. ±4 ± 6.0	0.556
High ANP, n, %	111(79.3%)	21 (75.0%)	90 (80.0%)	0.820
Low total iron, n, %	106(75.7%)	21 (75.0%)	85 (75.0%)	0.751
Anaemia, n, %	106(75.7%)	22 (78.0%)	84 (75.0%)	0.693
ID, n, %	84(60.0%)	16 (57.0%)	68 (60.0%)	0.663
CKD, n, %	82(58.6%)	24 (85.0%)	58 (51.0%)	0.038*
Comorbidities, n	5.9±1.9	7.9 ± 1.5	5.4 ± 1.9	0.000*

* Statistically significant

Values are expressed as mean ± standard deviation or number (percentage).

Key to **the** table: DM- diabetes mellitus, SBP- systolic blood pressure, DBP- diastolic blood pressure, HR- heart rate, BMI- body mass index, PCV- packed cell volume, Hb-haemoglobin, AF- atrial fibrillation, FBS- fasting blood sugar, LAD- left atrial dimension, RVD- right ventricular dimension, EF- ejection fraction, PCV- packed cell volume, LVMI- left ventricular mass index, KCCQ- Kansas City cardiomyopathy score, 6MWT- six minutes' walk test, NYHA- New York Heart Association, HTN- hypertension, ANP- atrial natriuretic peptide, ID- iron deficiency, CKD- chronic kidney disease.

N.B. Intracardiac clots **refer** to clot(s) in at least one area of the heart chambers

The clinical, demographic, and functional characteristics of HF subjects with a diagnosis of AF compared to those with normal sinus rhythm are also shown in Table 1. HF subjects with AF were not significantly different in age, systolic blood pressure, diastolic blood pressure, right ventricular internal dimension, packed cell volume, and gender distribution from those with sinus rhythm. They were also not significantly different in the frequency of hypertension, high serum atrial natriuretic peptide, low total iron, anaemia, and iron deficiency when compared with those with sinus rhythm. Even though HF subjects with AF were younger than those with sinus rhythm as shown in Table 1, the difference did not reach statistical significance. Fasting blood sugar (8.4± 2.0 vs. 5.4 ± 0.8 mmol/l, p <0.05), left atrial internal dimension (54.5 ± 8.5 vs. 45.5± 11.6 mm, p <0.05), and left ventricular mass index (103.1 ± 42.7 vs. 60.2 ± 44.7 g/m^2^, p <0.05) were significantly higher among HF subjects with AF than those with sinus rhythm. Ejection fraction was significantly lower among HF subjects with AF compared with subjects with sinus rhythm (37.7± 5.4 vs. 44.5 ± 10.1% respectively, p <0.05).

The mean six minutes walk test distance was significantly lower among HF subjects with AF compared to those with sinus rhythm (171.1±88.9 meters vs. 225.8 ± 102.1 meters respectively, p <0.05) as stated in Table 1. Pulmonary hypertension, intracardiac clots, and kidney dysfunction (estimated glomerular filtration rate <60 ml/min) were more frequent among HF subjects with AF than those with sinus rhythm. Severe HF as evaluated with the New York Heart Association (NYHA) classification (NYHA class IV) more frequently occurred among HF subjects with AF than those with normal sinus rhythm. HF subjects with AF had a higher frequency of clusters of comorbidities than those without AF as shown in Table 2 (7.9 ± 1.5 vs. 5.4 ± 1.9, p <0.05).

**Figure 1:**
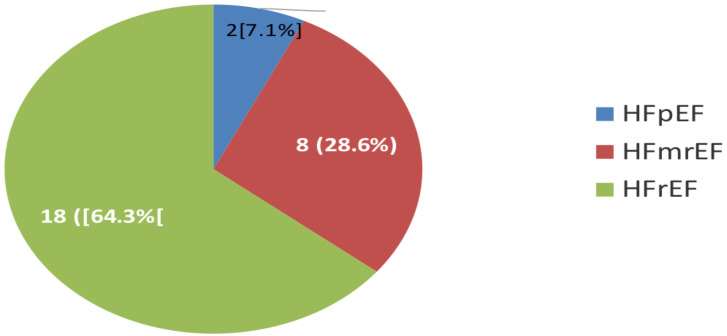
Distribution of heart failure phenotypes among subjects with atrial fibrillation Key: AF= atrial fibrillation, HF= heart failure, HFpEF= heart failure with preserved ejection fraction, HFmrEF= heart failure with mid-range ejection fraction, HFrEF= heart failure with reduced ejection fraction P <0.05: statistically significant

AF was more prevalent in patients with heart failure with reduced ejection fraction (HFrEF) compared to heart failure with preserved ejection fraction (HFpEF) and heart failure with mid-range ejection fraction (HFmrEF) as shown in Figure 1. The left atrial dimension was the largest among HFrEF patients as also shown in Figure 2, and this difference was statistically significant.

Table 2 highlights the various clinical and laboratory parameters of the study participants based on the presence or absence of AF. The prevalence of anaemia was high between the two groups even though relatively higher among participants with AF, but the difference was not statistically significant. AF in HF also occurred more often among middle-aged and elderly subjects, but this finding was also not statistically significant. The high atrial natriuretic peptide was not significantly different between the two groups, likewise gender distribution. The prevalence of AF increased as the severity of HF increased, as shown in Table 2 and Figure 3. Likewise, AF was more prevalent in HFrEF compared to other HF phenotypes.

## Discussion

The clustering of AF and HF portends serious functional, clinical, and prognostic challenges. Identifying the relationship and bidirectional impact between the two clinical syndromes is important to ascertain the risk and mitigate the additional risk of morbidity and mortality from cardiovascular disease. This study revealed that AF is found in at least one-fifth of HF patients. It is most likely that the frequency would have been more than this if long-term (Holter) Electrocardiography (ECG) had been done. This is in contrast with the earlier assertion that cardiac arrhythmias such as AF may be very rare among Africans(6,8–10). The epidemiology of AF in HF may be affected by several demographic and clinical variables(6,15). The prevalence of AF rises as the severity of HF (based on NYHA classification) increases, with the highest prevalence among those in NYHA class IV (6,15). This prevalence is similar to what has been described (prevalence rate of 15–45%) in other populations (18–22). Similarly, more of these patients were in the NYHA IV category in line with other reports. Nigeria and other sub-Saharan African countries are facing a double burden of infectious and non-communicable diseases including stroke, the burden of which may be increasingly related to the dual clustering of AF with HF(23). Most of our study participants had a history or diagnosis of hypertension, corroborating the fact that hypertension is still responsible for most cases of HF in Africa. With the increasing burden of HF across Africa, it is expected that the burden of AF may also increase and impact the clinical profile, prognosis, and treatment of chronic HF subjects (9,23–24).

The mortality rate among Africans with HF has been reported to be at least twice the world average and about four times that of South America. A potential reason for this, among others, is the additionally worse comorbidity profile, which AF contributes to (25–26). The prevalence of AF among the HF cohort in this study is however different from that of some other populations where AF in HF has been mostly related to HFpEF phenotype. Among HFpEF patients, the prevalence of AF has been reported to be 20–40% (27–29). In this study, AF was more frequent among subjects with HFrEF and those with advanced HF as evaluated by the NYHA classification. Studies linking AF with HFpEF have suggested that almost two-thirds of HF patients may develop arrhythmias during the course of the disease (30). The reason for this discrepancy may be connected to several factors. First, it appears that HFrEF patients present more frequently in the hospital in sub-Saharan Africa even though there is an increasing report of HFpEF in sub-Saharan Africa (6,9). Secondly, the aetiology of AF may be linked to several underlying aetiologies, whose prevalence in the two populations may vary and thus contribute to the different epidemiologies of AF among the various HF phenotypes (31). The clustering of AF more with HFrEF may also account for the exaggerated mortality among Africans as the combination of the two conditions portends a worse prognosis and advanced HF symptomatology. This conclusion may be indirectly inferred from the association with poor conventional markers of morbidity and mortality in HF in this study, including lower ejection fraction, higher chamber dimensions, and increased left ventricular mass index among others. The varied pattern of AF between HFpEF and HFrEF and among Caucasians and Africans may also be related to the pattern of aetiology of HF between the two populations and some genetic predisposition. Risk factors such as hypertension, diabetes mellitus, and ischemic heart disease predominate in HFpEF, and hypertension, valvular cardiomyopathy, dilated cardiomyopathy, and peripartum cardiomyopathies are important aetiologies of HF among Africans (32,33). The findings of higher prevalence of AF in HFrEF compared with HFmrEF and HFpEF in this study may also be linked to greater left atrial dilatation and not hypertension as shown in Figure 2 above.

Other authors have also suggested that AF is more prevalent among men than women (34). This study however found no association of gender with AF in HF subjects, contrary to other reports that have reported such a link (11,14). This may be related to regional variation and relatively small sample size among others.

**The prognostic implication and clinical correlation of clustering of AF with HF**: This study showed several interesting findings when AF coexisted with HF. Subjects with AF and HF had poorer conventional indices of functional status, mortality, and prognosis when compared to age- and gender-corrected HF cohorts with sinus rhythm. Subjects with coexisting AF and HF had significantly reduced ejection fraction and a higher proportion of kidney impairment, pulmonary hypertension, intracardiac clots, and associated comorbidities than those without AF. The left atrial dimension was also significantly higher among those with AF. The six minutes walk test was significantly lower among HF subjects with AF than those with sinus rhythm. This revealed that the presence of AF in HF patients is associated with poorer prognosis and functional status compared to when patients have sinus rhythm. Many of these factors are conventional markers of prognosis in HF and have been shown, in large populations, to be highly predictive of morbidity and mortality (35–37). It is established that the combination of AF and HF presents a worse prognosis than either alone (6,9,21,27). It is also evident from this study that HF when associated with AF among Nigerians is associated with a poorer predictive outcome. The presence of AF may encourage further atrial dilatation and increased frequency of cardiac thromboembolism as found in this study where intracardiac clots predominated among HF subjects with AF. The unusually high prevalence of cardiac thromboembolism may be related to the methods used in this study. Transthoracic echocardiography could overestimate intracardiac clots which could have been further confirmed with other diagnostic modalities such as cardiac magnetic resonance imaging or transesophageal echocardiogram which are however not available in the study center. Subjects in this group also had a higher prevalence of pulmonary hypertension, which is particularly associated with worse outcomes in HF (38,39).

HF subjects who had AF in this study had poorer functional profiles than those with sinus rhythm and are most likely at greater risk of mortality than those with sinus rhythm (3,5,9). Other authors have shown that AF in HF is associated with poorer quality of life compared to HF with sinus rhythm (16,22,29). This study also showed that AF appears to be more common with increasing age although this observation did not achieve statistical significance, probably because of the relatively small sample size. AF and HF are both disproportionately more frequent with increasing age (40). This is related partly to the increasing burden of risk factors for both conditions with increasing age. With increasing lifespans across Africa in recent decades, it is expected that the burden of cardiac arrhythmias, including AF, and HF may contribute to the non-communicable disease profile that has been predicted to increase substantially in the decades to come (23,25,26).

There is a lot of interest in the pathophysiology of AF in HF in recent times. The factors responsible for the initiation and propagation of AF in HF are multiple, complex, and interrelated. AF and HF also share common aetiologic factors. Therefore, their interaction can be related to the additional and multiplicative impact of these predisposing factors on the quality of life, symptomatology, and clinical outcomes of AF patients. The association of left atrial dilatation and AF is well described in the literature. Left atrial dilatation is both a cause and an effect of AF (41). The thin-walled left atrium cannot cope with increased left ventricular filling pressure (end-diastolic pressure) often due to hypertrophy caused by systolic and/or diastolic dysfunction in HF. This induces fibrotic changes in the atrial myocardium and, coupled with electrolyte and neurohormonal imbalance in HF syndromes and intrinsic myocardial disease often leads to the initiation and propagation of AF**.** The mechanisms by which HF can lead to an arrhythmogenic atrial substrate, including fibrillation, are elevated left-sided filling pressures, mitral regurgitation, atrial enlargement, interstitial fibrosis, and electromechanical remodeling (13,14). Others include dysregulation in the renin-angiotensin-aldosterone system, sympathetic nervous system activation, and intravascular calcium handling changes (14,15). On the other hand, AF can lead to HF through many mechanisms, including loss of atrial systole, functional mitral and tricuspid regurgitation, tachycardiomyopathy, and reduced ventricular diastolic filling time (16). The association of heart failure with many other comorbidities increases the risk of morbidity and mortality even among children. In a cohort in Ethiopia, Anemia and renal cases contributed to 50(23.1) and 12(5.6%) of HF cases reported in a tertiary centre (42).

This study has some limitations. First is the fact that long-term ECG such as Holter ECG was not done to identify patients with paroxysmal and persistent AF. Evaluating this would have increased the observed prevalence of AF among HF subjects: the single ECG done would most likely have underestimated the burden of AF among HF subjects, and this is a major limitation of this study. There is also the need for a longitudinal prospective study to document the prognostic association and differences in outcome and quality of life in HF subjects with AF compared to those without AF. We could not evaluate these associations and differences due to the design of the study. The relatively small sample size also limits the generalizability of this cross-sectional review: this was a hospital-based study, and its findings may therefore not be completely representative of the general population.

Conclusively, this study showed that AF is commonly associated with heart failure, especially HFrEF, in Nigerians. It may be associated with poor quality of life, poorer function and temporal profile, and increased risk of cardiac thromboembolism as evidenced by the higher prevalence of intracardiac clots found in this study. It is associated with advanced HF and may be closely related to left atrial dilatation and more with HFrEF among HF phenotypes among Africans with HF.

## Figures and Tables

**Table 2:** Factors associated with atrial fibrillation in heart failure subjects

Variable		AF (n=28)	Sinus rhythm (n=112)	P value
BMI	Normal BMI	15	53	0.235
	Overweight	5	25	
	Obesity	8	18	
AGE GROUP	Young	4	16	0.808
	Middle-aged	11	37	
	Elderly	13	59	
ANP GROUP	High ANP	21	90	0.603
	Normal ANP	7	22	
NYHA CLASS	I/II	7	40	0.047*
	III	7	43	
	IV	14	29	
HF PHENOTYPE	HFpEF	2	20	0.019*
	HFmrEF	8	38	
	HFrEF	18	54	
GENDER	Male	15	45	0.200
	Female	13	67	

* Statistically significant

Key to **the** table: BMI- body mass index, PULM HTN- pulmonary hypertension, ANP- atrial natriuretic peptides, NYHA- New York Heart Association, HF- heart failure, HFpEF- heart failure with preserved ejection fraction, HFmrEF- heart failure with mid-range ejection fraction, HFrEF- heart failure with reduced ejection fraction, AF- atrial fibrillation
